# Sustainable Aluminosilicate Coatings from Palm Oil Waste for Enhanced Thermal and Microstructure Properties

**DOI:** 10.3390/ma18040821

**Published:** 2025-02-13

**Authors:** Mohd Afdhal Shamsudin, Faizal Mustapha, Mohd Na’im Abdullah, Mazli Mustapha

**Affiliations:** 1 Faculty of Technology and Mechanical Engineering, Universiti Teknikal Malaysia Melaka, Melaka 76100, Malaysia; afdhal@utem.edu.my; 2 Department of Aerospace Engineering, Faculty of Engineering, Universiti Putra Malaysia, Serdang 43400, Malaysia; naimabdullah@upm.edu.my; 3 Department of Mechanical Engineering, Universiti Teknologi PETRONAS, Seri Iskandar 32610, Malaysia

**Keywords:** palm oil fuel ash, geopolymer, fire resistance, thermal insulation, sustainable materials, microstructural analysis

## Abstract

Geopolymers have emerged as promising materials for their superior thermal and mechanical properties, offering sustainable alternatives to conventional coatings. This study investigates the potential of Palm Oil Fuel Ash (POFA) as a raw material for fire-resistant geopolymer coatings. Through the optimization of POFA-to-alkaline activator (AA) ratios, NaOH concentrations, and curing temperatures, POFA-based coatings were synthesized and applied to mild steel substrates. Fire resistance testing revealed that the optimal formulation (0.35 POFA ratio, 8 M NaOH concentration, and curing at 65 °C) achieved a temperature at equilibrium (TAE) of 151.2 °C, significantly outperforming other compositions by reducing heat transfer during fire exposure. Thermal imaging and SEM analysis demonstrated that the optimized coating (GP-POFA8) exhibited a more uniform and stable intumescent layer, leading to lower peak temperatures (909 °C) compared to less optimized samples. Thermogravimetric Analysis (TGA) further confirmed that GP-POFA8 retained approximately 80% of its original mass at temperatures beyond 600 °C, highlighting its superior thermal stability. These findings underscore the potential of POFA-based geopolymers as effective, eco-friendly solutions for fire-resistant applications in construction and industrial sectors, contributing to sustainable waste management.

## 1. Introduction

The thermal and mechanical properties of materials are critical in determining their suitability for various engineering applications, especially in the aerospace, automotive and building industries. These properties directly influence the durability, safety, and performance of structures and components subjected to extreme thermal conditions [1,2]. In high-temperature environments, selecting materials that provide effective thermal insulation, fire resistance and structural integrity is crucial to ensure longevity, energy efficiency, and safety in various industrial sectors.

One of the most widely used thermal coating materials is silica aerogel, which has gained attention as a thermal barrier coating (TBC) in aerospace, automotive, and building applications due to its low thermal conductivity, lightweight nature, and high thermal stability [3,4]. This porous nanostructured material is composed of silica nanoparticles, forming a low-density structure with low thermal conductivity, high surface area, and a low dielectric constant [5,6]. Iswar et al. (2019) highlighted that pure silica aerogel can achieve thermal conductivity values as low as 0.01 W/mK at 25 °C, making it one of the most effective insulators available [7]. Similarly, He et al. (2016) highlighted that the ultralow thermal conductivity of silica aerogels is ideal for aerospace insulation systems, where weight reduction is a priority alongside thermal management [8]. In automotive applications, silica aerogel has been explored for the thermal insulation of battery packs, exhaust systems, and engine components [9]. Additionally, silica aerogel is integrated into fire-resistant paint, panels, windows, and insulation systems to enhance energy efficiency and fire safety in building applications [10]. Despite these advantages, silica aerogel faces significant challenges, including high production costs [11], mechanical fragility [12], and potential health risks due to its irritant nature [6]. These limitations have driven research toward alternative materials that can deliver similar thermal performance while addressing these drawbacks.

In response to these challenges, fireproof cement material and geopolymers have emerged as promising alternatives, offering environmental and performance benefits for thermal barrier applications [13,14]. Geopolymers, synthesized from aluminosilicate sources such as fly ash, slag, and kaolinite, are known for their low environmental impact and high thermal stability [15]. Research by Provis and van Deventer (2007) demonstrated that geopolymers could enhance the durability of structures exposed to high temperatures, making them suitable for applications such as coatings [16]. Traditional geopolymers have proven effective in providing thermal protection, as Saxena et al. (2017) demonstrated that fly ash-based geopolymers can maintain structural integrity even at temperatures exceeding 800 °C [17]. Sarcinella & Frigione (2023) highlighted the growing demand for sustainable coatings driven by environmental concerns, regulatory pressures, and the need to reduce reliance on petroleum-based materials. Their study emphasizes the increasing use of bio-based and waste-derived materials, such as plant-derived resins, cellulose nanocrystals, and agricultural byproducts, in the development of eco-friendly protective coatings [18]. To further improve sustainability and reduce reliance on conventional materials, recent studies have explored the use of agricultural byproducts, such as rice husk ash (RHA) [19], Palm Oil Fuel Ash (POFA) [20], and sugarcane bagasse ash (SCBA) [21] in geopolymer synthesis.

Among these alternative sources, Palm Oil Fuel Ash (POFA) presents a particularly compelling option. As a byproduct of the palm oil industry, POFA is abundant in many regions, offering a sustainable raw material for geopolymer synthesis. Utilizing POFA contributes to effective waste management while reducing reliance on cement, fire-resistant coatings, and insulation materials. As a partial replacement for Ordinary Portland Cement (OPC), POFA lowers CO_2_ emissions from cement production. Its high silica content enables its use in geopolymer composites, mortar, and lightweight panels, reducing the need for quarried sand, clay, and limestone [20]. Additionally, POFA-based coatings offer a sustainable alternative to ceramic-based insulators and synthetic fireproofing materials. By repurposing this agricultural byproduct, industries can cut material costs, minimize environmental impact, and support circular economy practices in green construction. According to Abdullah et al. (2024), POFA-based geopolymers have shown comparable or even superior thermal and mechanical properties to those derived from conventional aluminosilicates, making them suitable for high-temperature applications [20]. Further, Salih et al. (2022) emphasized that POFA geopolymers offer enhanced fire resistance due to their ability to form a stable matrix, which is critical for maintaining structural stability during fire exposure [22]. These studies suggest that POFA geopolymers can be an effective alternative to traditional materials like silica aerogel, providing a balance between performance and sustainability.

Despite the promising attributes of POFA-based geopolymers, a significant research gap remains in optimizing their synthesis parameters to enhance thermal insulation and fire resistance properties [23]. The effectiveness of POFA-based geopolymers depends on key activation parameters, including the alkaline activator concentration, POFA-to-alkaline activator (AA) ratio, NaOH molarity, and curing conditions. An improper balance can compromise thermal resistance and mechanical integrity, limiting practical applications. Additionally, while geopolymers generally exhibit good fire resistance, achieving a stable intumescent layer in POFA-based coatings remains a challenge. The influence of these parameters on the microstructure and thermal stability of the coatings has not been extensively studied.

Although previous research has established the feasibility of POFA-based geopolymers, systematic optimization studies to maximize their fire resistance and thermal insulation are still lacking. This study addresses these gaps by systematically optimizing the synthesis parameters, including the POFA-to-AA ratio, NaOH molarity, and curing temperature, to enhance fire resistance and thermal stability. Such optimization is crucial for positioning POFA-based geopolymer coatings as viable alternatives to high-performance thermal coatings like silica aerogel. By precisely adjusting these variables, this research demonstrates how targeted optimization can significantly improve material performance, facilitating real-world applications. Demir and Derun (2019) suggested that techniques such as Response Surface Methodology (RSM) provide valuable insights into how synthesis parameters affect thermal performance [24], reinforcing the importance of systematic optimization.

This study aims to explore the potential of POFA-based geopolymers as sustainable thermal coatings, focusing on optimizing their synthesis process to enhance thermal insulation and fire-resistant properties. Using Response Surface Methodology (RSM) for systematic optimization, this study investigates the effects of POFA-to-alkaline activator (AA) ratio, NaOH molarity, and curing temperature on the thermal performance of the coatings. Fire resistance tests were conducted by applying the POFA-based geopolymer coating onto a mild steel plate with dimensions 100 mm × 100 mm × (1.0 ± 0.3 mm) to assess its heat resistance. Additionally, thermogravimetric analysis (TGA), scanning electron microscopy (SEM), and thermal imaging were used to evaluate the thermal behavior, microstructural characteristics, and stability of the optimized formulations. Specifically, it seeks to demonstrate that POFA-based geopolymers can offer a viable alternative to silica aerogel and other traditional coatings while addressing the persistent need for greener materials in high-temperature applications. By advancing the understanding and optimization of POFA-based geopolymers, this study contributes to broader global efforts to develop sustainable materials, reduce industrial carbon footprints, and promote circular economy practices in material manufacturing.

## 2. Materials and Methods

### 2.1. Raw Materials

A total of 10 kg of raw POFA in the form of powder was obtained from S&S Coco Trading (Padang Besar, Perlis, Malaysia). The POFA was pulverized and sieved through 50 μm to remove impurities and obtain a finer particle size of POFA. The chemical compositions of POFA are shown in Table 1.

### 2.2. Preparation of POFA-Geopolymer Coating

Solid sodium hydroxide pellets (R&M Chemicals, Selangor, Malaysia) were dissolved in distilled water to obtain a sodium hydroxide (NaOH) solution. 6, 8 and 12 M of NaOH solution were prepared by diluting the sodium hydroxide pellets with distilled water according to Equation (1):

(1) $$\mathrm{Molarity}\;(M)=\frac{Moles\;of\;solute\;(n)}{Liters\;of\;solution\;(V)}$$

Since the molar mass of solid NaOH obtained from the supplier was 40.0 g, 1 L of water was required to obtain 1 M of NaOH solution. In order to obtain 6 M of NaOH solution, 120 g of NaOH pellet was dissolved in 500 mL of distilled water. The ratio of solid NaOH pellet to distilled water used was 0.04, which was applied to produce 8 M and 12 M of NaOH solution. The mass of NaOH pellet to dissolve in 500 mL distilled water to obtain 6 M, 8 M and 12 M of NaOH solution is shown in Table 2.

An activated alkaline solution of NaOH and sodium silicate solution (Na_2_SiO_3_) was prepared by mixing the two solutions until well combined. The ratio of the Na_2_SiO_3_ solution to the NaOH solution was kept constant at 2.5 by the mass of each solution to form the activated alkaline solution [25,26].

The ground POFA was combined with the activated alkaline solution at the designated ratio by mass of Palm Oil Fuel Ash (POFA): activated alkaline (AA). Three different ratios of POFA:AA were selected, which were 0.35, 0.45 and 0.55 by the mass of the POFA and activated alkaline (AA) solution to form the geopolymer POFA (GP-POFA) coating accordingly. The solutions were mixed using the same RW20 digital stirrer (IKA, Selangor, Malaysia) for 5 min until the solution became homogeneous. The geopolymer coating solution of activated alkaline (AA) and POFA appeared to be in dark brown color.

**Figure 1:**
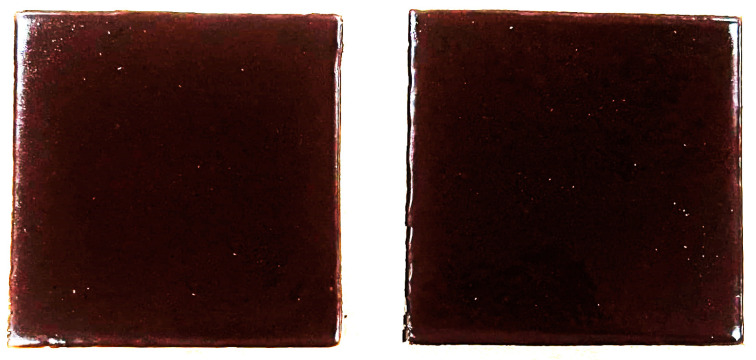
GP-POFA Samples.

The GP-POFA solution was spread evenly on a clean mild steel plate and left to cure for 24 h, as shown in Figure 1. The specimens were cured at three different temperatures, which were at ambient temperatures of approximately 28 °C, 45 °C and 65 °C [26,27,28]. For specimens cured at 45 °C and 65 °C, the coated substrates were placed in the oven for 24 h and stored at ambient temperature until testing. One layer of GP-POFA was applied to the mild steel until a ±0.3 mm coating thickness was attained.

### 2.3. Experimental Design and Analysis

**Figure 2:**
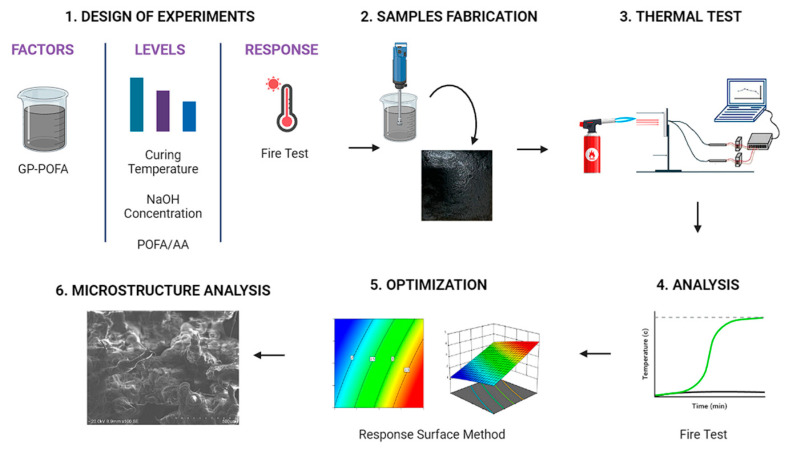
Overall flow of the experiment.

The flow of this experiment was assisted by using the Design of Experiment (DOE) in Design Expert software version 11 (Stat-Ease Inc., Minneapolis, MN, USA) to plan the experiment based on several important factors that may affect the result. Several important factors, namely the concentration of sodium hydroxide (NaOH), POFA: AA ratio and the curing temperature of the geopolymer coating, were considered in order to plan the experiment using DOE. Once the factors were determined, the optimization process was conducted by using the Response Surface Method (RSM) in Design Expert to produce the composition for each factor. This study focused on fabricating and testing natural aluminosilicate coatings, utilizing Palm Oil Fuel Ash (POFA) as a critical component. Figure 2 provides a visual overview of the comprehensive process involved in this study. This visual representation elucidates the sequential steps undertaken, from the initial stages of material selection and preparation through the fabrication process of the aluminosilicate coatings to the final phase of testing and analysis. The utilization of POFA in the coating process is a pivotal aspect of this methodology, underscoring the innovative approach to leveraging industrial by-products in material science applications.

For the RSM, the Box-Behnken design was selected for the three factors determined with only one replication to produce the number of runs for this experiment. The high and low level of each independent variable is set according to the selected ratios and concentration range as in Table 3. Since there are three independent variables based on the factors that were set for this experiment, the total number of runs generated by the RSM was 15 according to the levels of each factor that was selected.

### 2.4. Analysis of Variance (ANOVA)

Once the response surface design was generated, the data obtained from fire resistance testing, which consists of temperature at equilibrium (TAE) and time taken to reach equilibrium (TTAE) were included in the same worksheet and the response surface design was analyzed by selecting the responses, which are the TAE and TTAE from the fire resistance test. The response surface regression of the TAE and TTAE with the factors were obtained and the Analysis of Variance (ANOVA) was generated. ANOVA was employed to assess the significance of the factors influencing the TAE and TTAE in this study. ANOVA is a statistical method used to determine the contribution of each factor and their interactions to the variation observed in the response variable, in this case, TAE and TTAE. The quadratic model was selected for the analysis, considering its ability to capture non-linear relationships between the factors and TAE and TTAE.

### 2.5. Fire Resistance Test

The fire resistance test for the geopolymer-coated steel specimens was conducted following the UL 1709 standard [29], which simulates rapid temperature rise fire exposure. This standard is widely used in industrial and petrochemical settings, where structural steel must be protected from hydrocarbon fires that reach 1100 °C within 5 min. The test setup included a direct blow torch with butane gas, positioned 7 cm from the specimen, ensuring direct flame exposure. The flame temperature was monitored using a Fluke 51 II Wired Digital Thermometer (Fluke, Everett, WA, USA), maintaining a range between 700 and 1200 °C, mimicking extreme fire conditions.

Temperature measurements were recorded using an Agilent 34970A Data Acquisition System (Agilent, Santa Clara, CA, USA), which connected a thermocouple to the back of the specimen. This setup enabled real-time tracking of temperature at equilibrium (TAE) and time taken to reach equilibrium (TTAE), which are critical indicators of the coating’s fire resistance. A FLIR T1050sc thermal camera (Teledyne FLIR, Wilsonville, OR, USA) was used to capture thermal imaging data, allowing for analysis of heat distribution and peak surface temperatures.

To ensure effectiveness in fire protection, the coating was evaluated based on key fire resistance criteria. The ability to limit heat transfer is essential, as steel loses 50% of its strength at 600 °C and can collapse at around 1100 °C. The coating should provide fire protection for 30, 60, 90, or 120 min, depending on structural requirements. In this experiment, the fire resistance test was conducted for 30 min, assessing the coating’s ability to withstand prolonged heat exposure under extreme conditions. An intumescent behavior is also necessary, where the coating expands and forms an insulating char layer that prevents direct flame exposure and reduces heat transfer.

In terms of thermal stability and durability, the coating should maintain integrity up to 800–1200 °C, ensuring long-term protection in extreme heat conditions. The test is conducted for 30 min to observe the formation of the intumescent coating on the coated mild steel plate [30]. A total of 15 specimens were tested to obtain the temperature at equilibrium (TAE) and the time taken for the specimen to reach equilibrium (TTAE). During the fire-resistance test, thermal images of the samples are recorded using FLIR T1050sc (Teledyne FLIR, Wilsonville, OR, USA) to analyze the distribution of heat across the surface of the coated mild steel.

### 2.6. Material Characterization and Microstructure Analysis

Samples were analyzed using two important analyses, which were X-ray diffraction (XRD) (Philips, Malvern, UK), and scanning electron microscopy (SEM) (Hitachi, Tokyo, Japan). XRD was used for phase identification of the GP-POFA sample. The GP-POFA sample residues of approximately 5 mg to 10 mg were analyzed using a Philips PW3050/60, which records the intensity of the reflected X-rays rotating at each angle 2theta (2θ) range from 20° to 80°. PANalytical X’Pert HighScore software version 4.9 (Malvern Panalytical Ltd., Malvern, UK) was used to identify the phase, diffraction pattern analysis, and pattern treatment of the diffraction data obtained from the XRD instrument. This analysis is to ensure that the produced GP-POFA from the different POFA/AA ratio, NaOH molarity and curing temperature processes is still in amorphous form. Scanning Electron Microscopy (SEM) was used to analyze the external morphology, including the surface texture and the char layer formation on the specimens. SEM imaging was performed using a Hitachi S-3400N (Hitachi, Tokyo, Japan) at the Microstructure Characterization Laboratory (MCL), Universiti Putra Malaysia, to examine the microstructure of the char layer formed on the geopolymer coating after the fire test.

### 2.7. Thermogravimetric Analysis

Samples were further analyzed by using Thermogravimetric Analysis (TGA) and Derivative Thermogravimetry (DTG) to determine the thermal properties of both samples. The analysis was conducted under nitrogen gas at a heating rate of 10 °C/min, covering a temperature range from 50 °C to 1000 °C. A critical factor in this assessment is mass retention, as an ideal fire-resistant coating should retain at least 50–80% of its mass beyond 600 °C, which indicates strong resistance to thermal decomposition. The DTG curves were used to identify the specific decomposition stages and peak temperatures, providing additional insights into the thermal degradation behavior of the samples. This combination of TGA and DTG allows for a more comprehensive evaluation of the coatings’ thermal stability and fire resistance performance, highlighting the impact of their composition and microstructure on their thermal properties.

## 3. Results and Discussions

The complete design matrix and response values of the time taken to reach equilibrium (TTAE) and temperature at equilibrium (TAE) are given in Table 4.

### 3.1. Analysis of Fire Resistance Test Using ANOVA

The ANOVA table for the quadratic model in Table 5 helps analyze the effect of different factors and their interactions on the response variable, TAE. The analysis indicates that the overall model is highly significant, with a *p*-value less than 0.0001 and an F-value of 243.52, suggesting that the model effectively captures a significant portion of the variation in TAE. This means that at least one term in the model contributes substantially to explaining the response.

Among the individual factors, A-POFA/AA displays a strong influence on TAE, evidenced by its *p*-value of 0.0001 and an F-value of 114.54, indicating its significant contribution to the variation in TAE. B-NaOH molarity, while less influential than POFA/AA, still shows a notable impact with a *p*-value of 0.0386 and an F-value of 7.77, signifying a significant effect though relatively modest compared to other factors. The most pronounced influence comes from C-Curing, with a *p*-value of less than 0.0001 and an F-value of 189.45, highlighting the importance of curing conditions in determining TAE.

The interactions between factors provide additional insights. The interaction between POFA/AA and NaOH molarity (AB) is not significant, with a *p*-value of 0.6476, suggesting that the combined effect of these two factors on TAE is minimal. In contrast, the interaction between POFA/AA and curing (AC) has a *p*-value of 0.0002 and an F-value of 85.71, indicating a significant effect on TAE when these factors are varied together. The most substantial interaction effect is observed between NaOH molarity and curing (BC), with a *p*-value of less than 0.0001 and an F-value of 1029.80. This interaction suggests that changes in NaOH concentration and curing conditions jointly have a significant impact on TAE. The analysis also reveals the presence of significant quadratic effects for each of the factors, including A^2^, B^2^ and C^2^, with *p*-values all less than 0.0001. This indicates that the relationships between these factors and TAE are not strictly linear but rather involve curvature, suggesting that the optimal levels of these factors are likely found somewhere in the middle of the experimental range rather than at the extremes.

The interaction between NaOH molarity and curing conditions is the most significant factor influencing TAE, while the quadratic terms suggest the importance of finding intermediate values for each factor.

The ANOVA table for the quadratic model analyzing TTAE in Table 6 provides insight into how different factors and their interactions influence this response. The overall model is statistically significant, with a *p*-value of 0.0106 and an F-value of 9.88. This indicates that the model can explain a significant portion of the variation in TTAE, implying that at least one of the model terms has a considerable impact on the response.

Among the individual factors, the influence of A-POFA/AA on TTAE appears to be relatively minor, as indicated by its *p*-value of 0.6844 and an F-value of 0.1857. This suggests that variations in the POFA/AA ratio do not significantly affect TTAE. Similarly, B-NaOH shows a limited effect on TTAE, with a *p*-value of 0.7518 and an F-value of 0.1116, indicating that changes in NaOH concentration are not strongly associated with variations in TTAE. C-Curing, while not statistically significant at the conventional level (*p*-value of 0.0610), has a more notable impact, reflected in its F-value of 5.80, suggesting that curing conditions could have a meaningful influence under different settings or slightly broader significance thresholds.

The interaction terms provide an additional understanding of how the factors work together to influence TTAE. The interaction between A-POFA/AA and B-NaOH (AB) is significant, with a *p*-value of 0.0086 and an F-value of 17.55. This indicates that the combination of POFA/AA and NaOH concentration plays a significant role in determining TTAE. The interaction between A-POFA/AA and C-Curing (AC) is also significant, with a *p*-value of 0.0151 and an F-value of 13.16, showing that changes in curing conditions have a different impact on TTAE depending on the POFA/AA ratio. However, the interaction between NaOH concentration and curing (BC) is not significant, with a *p*-value of 0.1187, indicating that their combined effect on TTAE is less pronounced.

The quadratic terms of the model reveal some non-linear relationships. The term for B^2^ (NaOH^2^) is significant, with a *p*-value of 0.0277 and an F-value of 9.44, indicating that the effect of NaOH concentration on TTAE is not purely linear but rather follows a curved pattern. The quadratic term for C^2^ (Curing^2^) is highly significant, with a *p*-value of 0.0023 and an F-value of 32.78, suggesting that curing conditions have a substantial non-linear impact on TTAE. In contrast, the A^2^ term (POFA/AA^2^) is not significant, with a *p*-value of 0.4214, indicating that the effect of POFA/AA on TTAE does not exhibit a strong curvature.

The ANOVA results suggest that the most influential factors on TTAE include the interaction between POFA/AA and NaOH and the non-linear effect of curing conditions. While the main effects of POFA/AA and NaOH are not individually significant, their interactions with other factors underscore their importance in the model.

The regression models can be used to calculate and analyze the effect of factors on the fire resistance performance of paint mixed with an FR additive. The equations are presented in terms of coded factors, and actual factors are useful to make predictions about the response for given levels of each factor.

The regression model for TAE and TTAE, respectively, expressed in terms of actual factors for a quadratic ANOVA with interactions can be written as:

Y_TAE_ = 186.864 + 7.73036 × A − 1.96214 × B + −9.92392 × C − 0.470142 × AB + 9.18753 × AC + 30.9965 × BC − 11.849 × A^2^ + 18.5288 × B^2^ + 11.2923 × C^2^

Y_TTAE_ = 650.746 − 19.1212 × A − 14.4473 × B + 106.648 × C + 248.953 × AB + 221.117 × AC + −111.614 × BC − 55.6276 × A^2^ + 225.212 × B^2^ + 366.792 × C^2^

### 3.2. Interaction Analysis of Factors Influencing Time at Equilibrium

In this section, the interactions between the factors AB (POFA/AA × NaOH), AC (POFA/AA × Curing temperature), and BC (NaOH × Curing temperature) were further analyzed on the influence on TAE. Understanding these interactions is crucial for determining how the combined effects of these factors influence the response variable and for identifying the optimal conditions that achieve desired outcomes.

**Figure 3:**
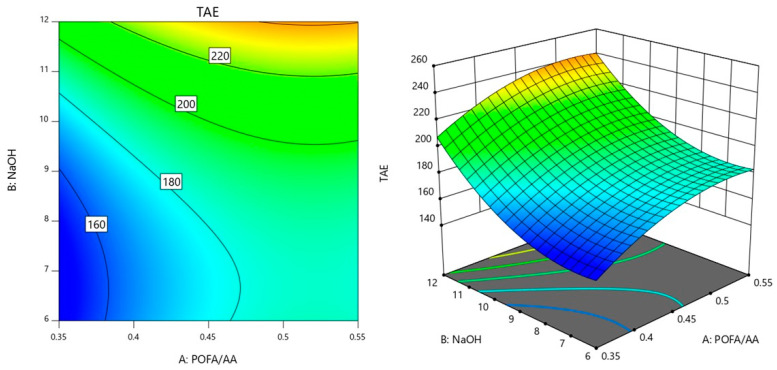
Contour plot of TAE variation with POFA/AA ratio and NaOH molarity.

In the interaction between the factors AB in Figure 3, the value of curing temperature was controlled at 65 °C because preliminary analysis indicated that this temperature resulted in the lowest observed TAE. By maintaining the curing temperature at this optimal level, the study aimed to further analyze the effects of varying POFA/AA and NaOH molarity conditions on the response while keeping the curing temperature constant to ensure consistency and highlight the interactions between the remaining factors under conditions that minimize TAE.

From Figure 3, it was observed that lower TAE values, around 160, are concentrated towards the lower left corner, where both POFA/AA and NaOH molarity are relatively low. This indicates that when using lower values of POFA/AA and NaOH molarity, the TAE tends to be minimized. The contours are closer together on the left side of the plot and spread out more on the right. This pattern suggests that changes in POFA/AA have a more significant impact on TAE when NaOH molarity is low, while the effect of increasing NaOH molarity becomes more noticeable at higher levels of POFA/AA. As a result, adjusting the NaOH molarity can have a more substantial effect on TAE when the POFA/AA is already high, leading to a steep increase in TAE values. This plot shows that TAE is highly sensitive to increases in both POFA/AA and NaOH. Higher concentrations of these factors lead to elevated TAE levels, while lower concentrations help keep the TAE values lower.

**Figure 4:**
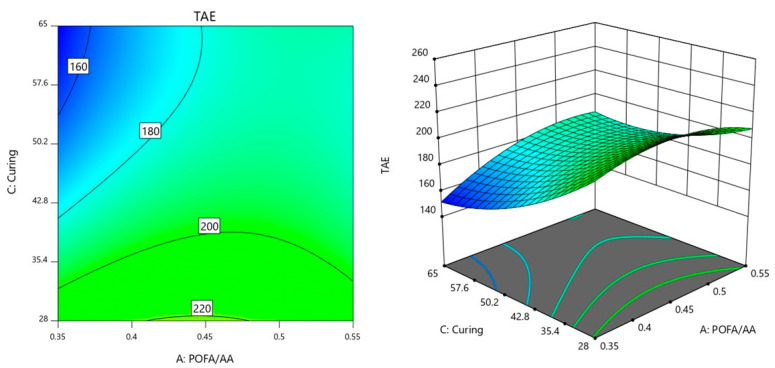
Contour plot of TAE variation with POFA/AA ratio and curing temperature.

The interaction between the factors of AC (POFA/AA × Curing temperature) is shown in Figure 4. For this interaction, the NaOH molarity was controlled at 8 M as preliminary analysis indicated that this concentration resulted in the lowest observed TAE. By maintaining NaOH molarity at this optimal level, the study aimed to further analyze the effects of varying POFA/AA and curing temperature conditions on the response while keeping NaOH constant to ensure consistency and highlight the interactions between the remaining factors under conditions that minimize TAE.

In Figure 4, in the bottom left corner of the plot, the TAE values are lowest, around 160, when both POFA/AA and curing temperature are at lower levels. This suggests that lower values of these factors tend to minimize TAE. As POFA/AA increases along the horizontal axis, the TAE values gradually rise, reaching levels like 180 °C and 200 °C, especially when the curing temperature is also increased along the vertical axis. The gradient of the contour lines shows that TAE increases more sharply with POFA/AA when the curing temperature is also relatively high.

Higher TAE values, such as 200 °C and 220 °C, are found in regions where POFA/AA is moderate to high, with curing temperatures closer to the upper middle range. This indicates that the combined effect of higher POFA/AA and higher curing temperatures is to increase TAE. The contour lines spread out in this region, suggesting a more gradual change in TAE as both factors reach their higher values.

**Figure 5:**
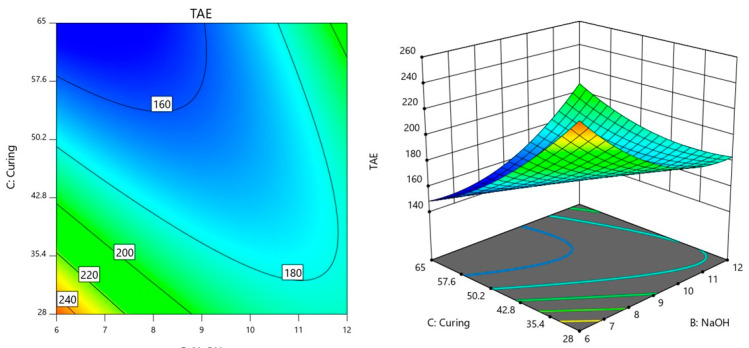
Contour plot of TAE variation with NaOH molarity and curing temperature.

The interaction between the factors of BC (NaOH × Curing temperature) is shown in Figure 5. For this interaction, the POFA/AA was controlled at 0.35 as preliminary analysis indicated that this concentration resulted in the lowest observed TAE. By maintaining POFA/AA at this optimal level, the study aimed to further analyze the effects of varying NaOH and curing temperature conditions on the response while keeping POFA/AA constant to ensure consistency and highlight the interactions between the remaining factors under conditions that minimize TAE.

The region with the lowest TAE value, around 160 °C, appears in the upper right part of the plot, where NaOH concentration is higher and curing temperature is also relatively high. This suggests that a combination of increased NaOH levels and higher curing temperatures tends to minimize TAE. As NaOH levels decrease, especially when paired with lower curing temperatures, the TAE values increase, reaching 200 °C and even 240 °C in the bottom left corner of the plot.

The curvature of the contour lines indicates a non-linear relationship between NaOH concentration and curing temperature. As NaOH concentration decreases and curing temperature lowers, the contours shift toward higher TAE values, indicating a strong interaction between these two factors in influencing the response. This means that decreasing both NaOH concentration and curing temperature leads to an increase in TAE, with the highest values occurring when both factors are at their lower limits.

In contrast, the contours are more spread out in the region where TAE is lower (around 160 °C), suggesting that changes in NaOH concentration have a more gradual effect on TAE when combined with higher curing temperatures.

### 3.3. Interaction Analysis of Factors Influencing Time Taken to Reach Equilibrium Temperature

Similarly, in this section, the interactions between the factors AB (POFA/AA × NaOH), AC (POFA/AA ×Curing temperature), and BC (NaOH × Curing temperature) were further analyzed on the influence on TTAE.

**Figure 6:**
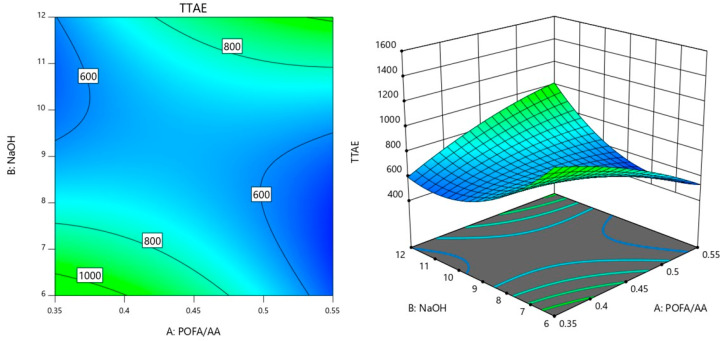
Contour plot of TTAE variation with POFA/AA ratio and NaOH molarity.

Figure 6 shows the contour plot illustrating the interaction between AB. The value of curing temperature was controlled at 45 °C as preliminary analysis indicated that this temperature resulted in the lowest observed TTAE. In the center of the plot, the response is lower, around 600 s, indicating that this region represents a combination of POFA/AA and NaOH that minimizes the TTAE. This suggests that there is a specific balance between these two factors where the interaction effect (AB) is most favorable for achieving a lower response value. The central area acts as a sort of valley, implying that adjusting the values of POFA/AA and NaOH to remain near this range results in a minimized TTAE.

As POFA/AA and NaOH values move away from this central region, the TTAE values increase, reaching 800 s and then 1000 s along the plot’s periphery. This increase indicates that when the balance between POFA/AA and NaOH is disturbed, either by increasing or decreasing one of the factors significantly, the interaction between them leads to higher TTAE values. This change in TTAE demonstrates that a precise adjustment of both factors is critical for maintaining a low response. The shape and curvature of the contour lines further illustrate how the interaction between POFA/AA and NaOH influences TTAE. In areas where the lines are closer together, small changes in either POFA/AA or NaOH result in rapid shifts in the response, highlighting a region where the interaction effect is highly sensitive. Conversely, where the lines are more spaced out, the response changes more gradually, suggesting that variations in POFA/AA or NaOH have less impact on TTAE. Maintaining the values of both factors near the central region results in lower TTAE values, while deviations from this balance lead to higher responses. The plot provides a clear visual guide for optimizing the combination of these two factors to achieve the desired outcome.

**Figure 7:**
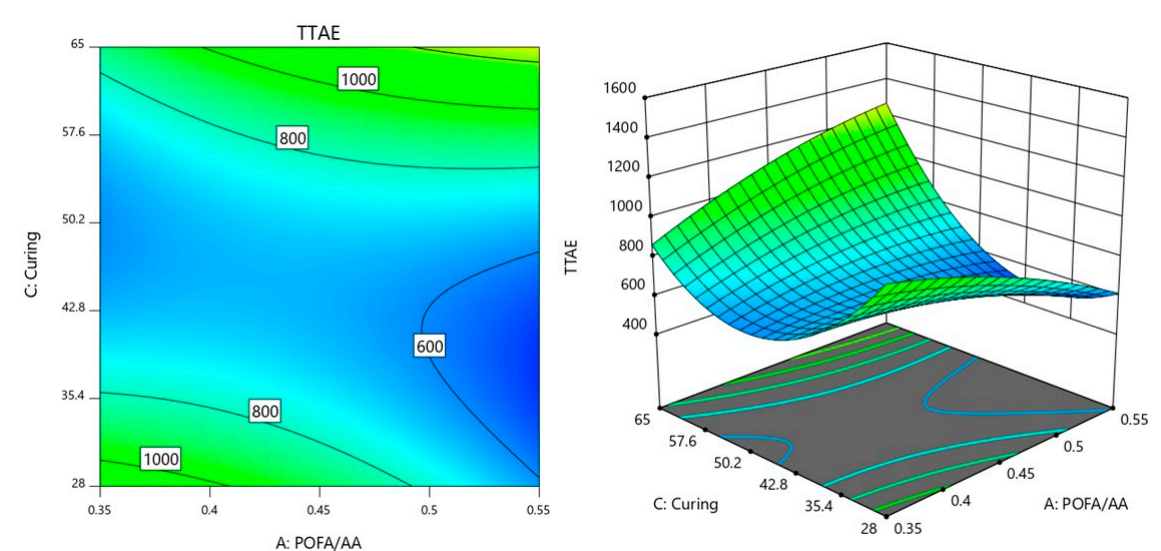
Contour plot of TTAE variation with POFA/AA ratio and curing temperature.

Meanwhile, the interaction between the factors of AC (POFA/AA × Curing temperature) is shown in Figure 7. For this interaction, the NaOH molarity was controlled at 8 M as preliminary analysis indicated that this concentration resulted in the lowest observed TTAE. In the Figure 7 contour plot, the TTAE value is minimized around the region labeled 600 s, which corresponds to lower POFA/AA ratios and lower to moderate curing temperatures. This suggests that the interaction between POFA/AA and curing temperature (AC) is favorable in this range, allowing for a reduction in TTAE when the balance between these two factors is optimized. As the POFA/AA ratio increases or the curing temperature rises beyond this range, the TTAE values increase, indicating that the interaction becomes less favorable for minimizing TTAE. At lower POFA/AA values, increasing the curing temperature leads to a gradual increase in TTAE, as shown by the spread of the contours between 600 s and 800 s. This suggests that at low POFA/AA ratios, the curing temperature has a more moderate impact on increasing TTAE. However, as POFA/AA values rise, the contours become closer together, indicating that changes in curing temperature at higher POFA/AA levels have a more pronounced effect on increasing TTAE.

Additionally, the regions where the contour lines shift from 600 s to 1000 s suggest that higher POFA/AA combined with increased curing temperatures leads to higher TTAE values. This indicates that when both factors are elevated, their combined effect (AC interaction) results in a less desirable outcome for TTAE, highlighting the importance of balancing these factors. The optimal region for minimizing TTAE lies where the balance between POFA/AA and curing temperature is maintained at lower values, while higher values of either factor lead to an increase in TTAE due to their interaction.

**Figure 8:**
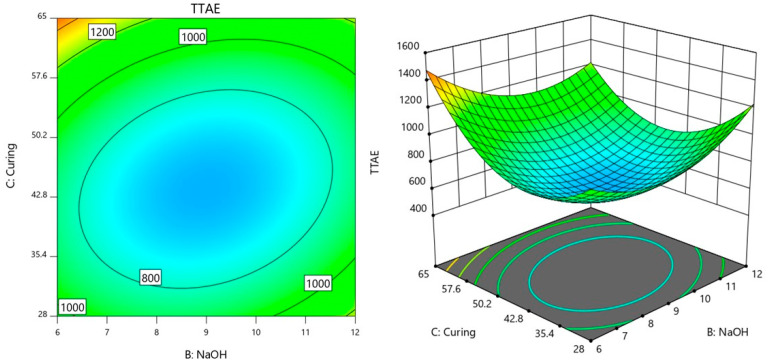
Contour plot of TTAE variation with NaOH molarity and curing temperature.

The contour plot in Figure 8 demonstrates the interaction between NaOH molarity (Factor B) and curing temperature (Factor C) in determining the TTAE response, with POFA/AA (Factor A) fixed at a value of 0.45. In the center of the plot, the TTAE values are lowest, around 800 s, forming an elliptical region that suggests an optimal balance between NaOH concentration and curing temperature. This area indicates that when NaOH and curing temperature are both kept near these central values, their interaction minimizes the TTAE. The elliptical shape suggests that there is a precise range of values for NaOH and curing temperature that provides the most favorable conditions for minimizing TTAE.

Outward from this central region, the TTAE values increase to 1000 s and eventually 1200 s. This indicates that when either NaOH molarity or curing temperature deviates significantly from their optimal range, the TTAE value rises. The interaction between NaOH and curing temperature (BC) becomes less favorable for minimizing TTAE as it moves away from the optimal balance. For example, higher NaOH levels combined with higher curing temperatures, or lower values for both, lead to higher TTAE values as indicated by the contours shifting to 1000 s and beyond. This reflects a strong interaction effect between NaOH and curing temperature, where the response is sensitive to adjustments in both factors. Achieving a low TTAE value requires maintaining NaOH concentration and curing temperature within the optimal central range. Deviations from this balance, either by increasing or decreasing the factors, result in less desirable, higher TTAE values.

The interactions between the factors POFA/AA (A), NaOH (B), and curing temperature (C) reveal how each pair of factors influences TTAE. The interaction between POFA/AA and NaOH (AB) shows that a balanced combination of these two factors is critical for achieving low TTAE values. Lower NaOH concentrations paired with lower POFA/AA ratios tend to produce lower TTAE while increasing both leads to a rise in TTAE. This relationship suggests that fine-tuning both factors together is key to controlling the response.

In the interaction between POFA/AA and curing temperature (AC), the plot indicates that maintaining moderate levels of both factors is crucial for minimizing TTAE. Low POFA/AA combined with lower curing temperatures helps to achieve lower TTAE values while increasing either factor raises the response. This interaction implies that the effects of curing temperature on TTAE are more pronounced when the POFA/AA ratio is higher, leading to a less favorable outcome if not balanced correctly.

The interaction between NaOH and curing temperature (BC) shows that the response is highly sensitive to changes in both factors. A specific balance between NaOH concentration and curing temperature is required to achieve the lowest TTAE. When both NaOH concentration and curing temperature are close to their optimal values, the TTAE reaches its minimum. However, deviating from this balance by either increasing or decreasing one of the factors results in higher TTAE values. This interaction emphasizes that precise adjustments in both factors are necessary for maintaining the desired response.

### 3.4. Optimization of the Responses and Model Validation

To optimize TAE based on the interactions of factors A (POFA/AA), B (NaOH molarity), and C (curing temperature), as observed in the previous contour plots, these factors need to be carefully balanced to minimize TAE. The AB interaction in Figure 3 suggests that lower TAE values are achieved when both POFA/AA and NaOH molarity are balanced at moderate levels. Thus, for optimal TAE, the POFA/AA in the range of 0.35 to 0.45 and NaOH molarity around 7 to 9 are favorable to minimize TAE. The contour plot for AC interaction in Figure 4 shows that TAE is minimized when POFA/AA is kept at moderate levels and the curing temperature is not too high. As curing temperature increases, especially at higher POFA/AA ratios, TAE tends to rise. For optimization, POFA/AA was set at 0.4 and the curing temperatures were in a moderate range of 35 to 45 °C. This balance helps maintain lower TAE by preventing the rise that occurs with high curing temperatures. Lastly, in the BC interaction plot (Figure 5), TAE is lowest when both NaOH and curing temperatures are at middle values. Extreme values of NaOH or high curing temperatures tend to elevate TAE. Thus, to achieve optimal TAE, NaOH levels were maintained around 7 to 9 while keeping curing temperatures in the range of 35 to 45 °C.

**Figure 9:**
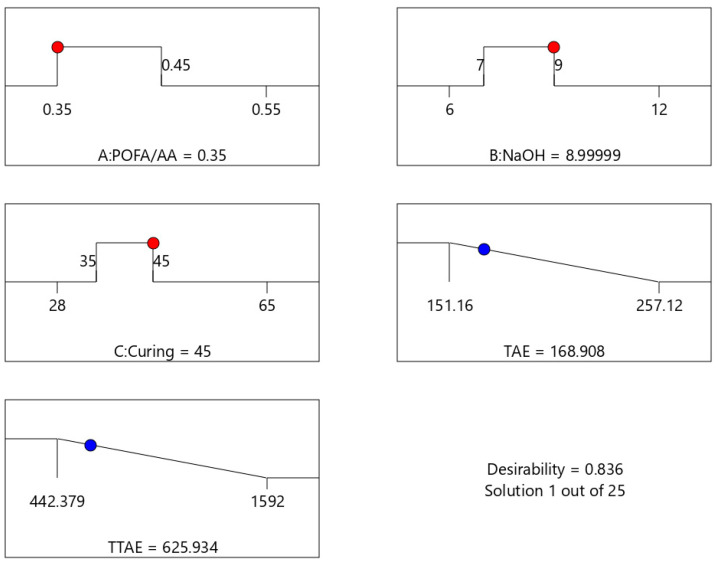
Optimum conditions and response for fire resistance properties.

Figure 9 illustrates the predicted optimum conditions for TAE and TTAE. The predicted optimum operating parameters influencing thermal properties were estimated at 0.35 POFA/AA, NaOH molarity of 8.99 and curing temperature of 45 °C. At these optimum conditions, the corresponding predicted TAE is 168.908 °C and the TTAE is 625.34 s.

Additional experimental runs at the optimized factor levels (POFA/AA of 0.35, NaOH concentration of 9, and curing temperature of 45 °C) were conducted to verify that the model’s predicted values match the actual experimental outcomes. This step ensures that the optimization results are reliable and can be reproduced. Table 7 shows the experimental validation for fire resistance properties. It was found that the average errors for the TAE and TTAE were well below 15% at 5.67% and 6.47%, respectively. It was concluded that the developed regression model established using this method was able to optimize the value of the responses.

### 3.5. Thermal Expansion Observation and Thermal Image Analysis

**Figure 10:**
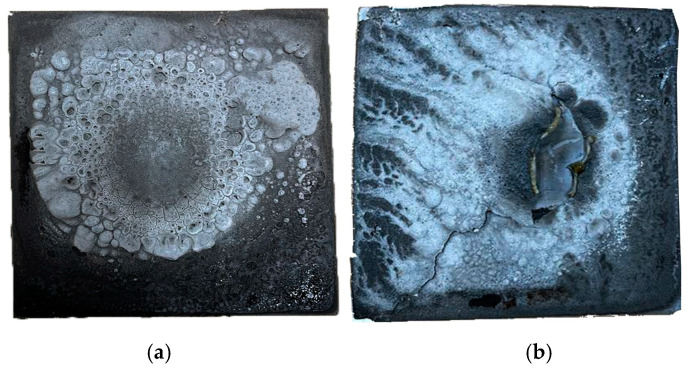
Post-fire test of sample; (**a**) GP-POFA8 and; (**b**) GP-POFA7.

Figure 10 shows the post-fire test results of two samples, GP-POFA8 (left) and GP-POFA7 (right). After being exposed to high flame temperatures, the samples exhibit different surface morphologies and damage characteristics, indicating variations in their fire resistance and thermal behavior.

The GP-POFA8 sample shows a relatively uniform swelling pattern with smaller, more evenly distributed bubbles across the surface. This uniformity suggests that the material underwent a controlled intumescent reaction, forming a consistent protective layer. The formation of this intumescent layer, which swells upon exposure to heat, can help insulate the inner structure from further temperature rise, reducing thermal degradation. This behavior aligns with the findings of Mariappan (2016), who observed that materials exhibiting a stable intumescent layer could better resist heat penetration and maintain their structural integrity under high temperatures [31]. In addition, higher NaOH molarity, as seen in the GP-POFA8 sample, results in a more complete dissolution of silica and alumina from the ash, which promotes the formation of a denser and more cohesive matrix. This dense matrix is better at resisting high temperatures due to its reduced porosity, which limits heat penetration. This is in accordance with a study by Alehyen et al. (2017), which found that higher NaOH molarity improves the compressive strength and thermal stability of fly ash-based geopolymers by creating a more consolidated structure [32].

In contrast, the GP-POFA7 sample shows a more irregular and severe surface deterioration, with larger cracks and a more noticeable collapse in the center. This suggests that the intumescent layer was less effective in providing uniform thermal protection. The cracks and damage indicate that the material’s thermal resistance was lower, which allowed heat to penetrate more deeply into the structure, leading to internal stresses and structural failure. This is consistent with studies by Wang et al. (2021), which found that a lack of effective intumescent layer formation can lead to thermal cracking and material failure when exposed to high temperatures [33].

Furthermore, research by Swanepoel and Strydom (2002) showed that higher curing temperatures are crucial for improving the mechanical and thermal properties of geopolymers, especially when paired with an appropriate NaOH concentration [34]. The combined effects of optimal NaOH molarity and optimal curing temperature in GP-POFA8 lead to a more effective geopolymer matrix with superior fire resistance.

Overall, GP-POFA8 demonstrates better thermal performance and fire resistance due to its ability to form a stable and protective intumescent layer, minimizing damage from high flame exposure, which aligned with the results obtained for TAE of 151.16 °C. Meanwhile, GP-POFA7 suffers from greater surface damage and cracking, which led to a higher TAE of 257.12 °C, indicating a less effective response to fire conditions. These observations suggest that the formulation of GP-POFA8 may include components that facilitate a more uniform intumescent reaction, making it a more suitable choice for applications requiring enhanced fire resistance.

**Figure 11:**
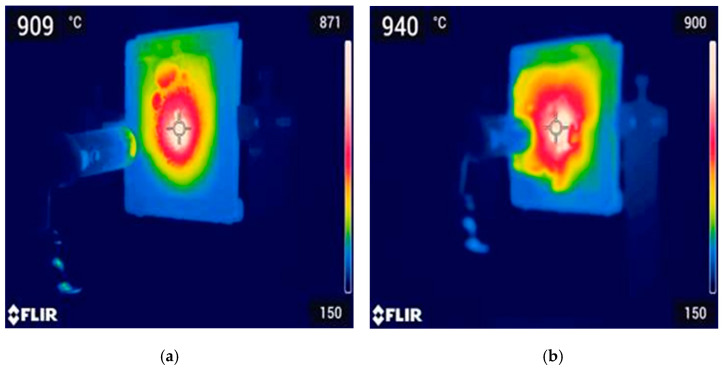
Thermal images of sample; (**a**) GP-POFA8 and; (**b**) GP-POFA7.

Thermal images were also taken during the fire resistance test, as shown in Figure 11. In the thermal images, GP-POFA8 displays a peak temperature of 909 °C, which is lower than the 940 °C peak observed for GP-POFA7. The lower peak temperature in GP-POFA8 suggests that this material is more effective at dissipating heat away from the surface, thus preventing excessive heat accumulation in localized areas. This characteristic is crucial for fire resistance as it reduces the likelihood of thermal stress and localized weakening when exposed to high temperatures.

On the contrary, GP-POFA7 retains a higher central temperature, indicating that it may absorb heat but is less efficient in dissipating it. The higher temperature at the surface implies that heat is not being conducted away as effectively, which could lead to a greater risk of structural damage when subjected to prolonged exposure to flames. This observation aligns with studies such as those by Lahoti et al. (2019), which emphasized that materials exhibiting lower surface temperatures during heat exposure are more suitable for fire-resistant applications, as they provide a better protective layer against heat penetration [35].

Additionally, research by Jhatial et al. (2021) highlighted that materials with good heat dissipation properties, particularly those incorporating industrial by-products like POFA and eggshell powder, tend to exhibit better fire resistance [36]. These materials create a barrier effect, reducing the temperature gradient through the material thickness, which helps maintain structural integrity under fire conditions. GP-POFA8’s ability to keep temperatures lower aligns with these findings, suggesting it has superior fire-resistant properties compared to GP-POFA7.

In conclusion, GP-POFA8 is likely more effective for fire resistance applications due to its better heat dissipation, as evidenced by the lower peak temperatures observed in the thermal imaging analysis. The ability of GP-POFA8 to manage heat more efficiently reduces the risk of thermal degradation during fire exposure, making it a suitable candidate for applications where fire resistance is critical. These findings are consistent with literature that underscores the importance of rapid heat dissipation in fire-resistant materials.

### 3.6. Material Characterization and Microstructural Analysis

The microstructure and thermal stability of geopolymer samples exhibiting the highest and lowest Time at Equilibrium (TAE) values are further analyzed using Scanning Electron Microscopy (SEM) and Thermogravimetric Analysis (TGA). These analyses provide better understanding of the material properties and behaviors under thermal stress.

**Figure 12:**
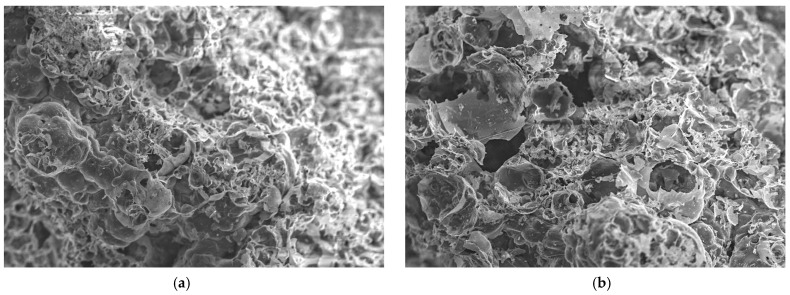
SEM images of sample of coating surface: (**a**) GP-POFA8 and; (**b**) GP-POFA7.

Figure 12 displays SEM micrographs of the coating surface where the intumescent formed on samples GP-POFA8 and GP-POFA7. The microstructural characteristics of two geopolymer samples were compared.

In the SEM image of Figure 12a GP-POFA8, the structure appears to be more compact, with relatively smaller and more evenly distributed pores. The denser microstructure suggests a higher degree of polymerization, which is typically associated with better mechanical strength and thermal resistance. A well-compacted matrix can enhance the material’s ability to act as an effective thermal barrier, as it minimizes heat transfer through the material. This observation is consistent with the findings of Rattanasak and Chindaprasirt (2009), who noted that higher NaOH concentrations during geopolymer synthesis promote more complete dissolution of silica and alumina, leading to denser microstructures [37]. The compact nature of GP-POFA8 indicates a lower level of interconnected porosity, which can contribute to its improved fire-resistant properties by limiting the penetration of heat through the material.

Meanwhile, the SEM image of GP-POFA7 in Figure 12b reveals a porous structure with larger voids with brittle intumescent layers and less uniform distribution of the geopolymer matrix. This brittle intumescent layer suggests a lower degree of polymerization, which can be attributed to factors like lower NaOH concentration or suboptimal curing conditions. The larger and more irregular brittle pores can lead to decreased thermal and mechanical stability, as they allow for more significant heat transfer and contribute to weaker structural integrity under stress. These findings align with the findings previously in Figure 10b where a severe surface deterioration, with larger cracks and a more noticeable collapse intumescent layer in the center, occurred in sample GP-POFA7. In addition, research by Frederickx et al. (2022), showed that geopolymers synthesized with lower NaOH concentrations tend to have more open and less cohesive microstructures, resulting in poorer mechanical properties and reduced resistance to thermal degradation [38].

The differences in porosity and matrix compaction between GP-POFA8 and GP-POFA7 directly impact their thermal insulation capabilities. The more compact microstructure of GP-POFA8 makes it a better candidate for applications that require enhanced fire resistance, as its dense matrix provides a more effective barrier against heat. In contrast, the higher porosity of GP-POFA7 suggests that it may be more susceptible to heat penetration and structural degradation under high-temperature conditions.

**Figure 13:**
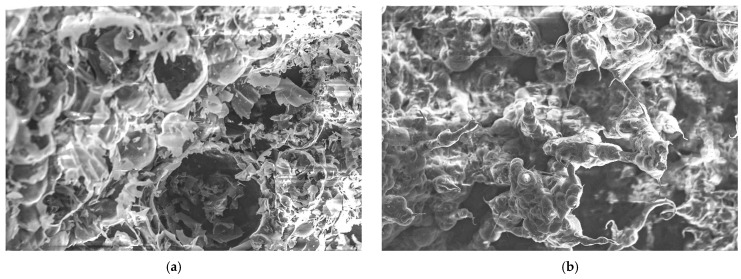
SEM images of sample inner surface: (**a**) GP-POFA8; and (**b**) GP-POFA7.

Figure 13 displays an SEM image of the coating inner surface where the intumescent formed on samples GP-POFA8 and GP-POFA7. SEM analysis can reveal the presence of unreacted particles or residual components within the geopolymer matrix after it has undergone the intumescent process. Unreacted particles may act as weak points within the material, affecting its mechanical strength and durability under thermal exposure [39].

As observed in Figure 13a, the GP-POFA8 sample in Figure 13a has compact with tightly bonded features and smaller voids. The microstructure shows smoother, more uniform surfaces, indicating a well-developed geopolymer matrix with fewer cavities. This suggests a higher degree of polymerization, where the reactive components have formed a continuous and dense network. The relatively smooth inner intumescent layer surfaces suggest effective bonding between the silica and alumina, resulting in a more cohesive structure that can better withstand heat.

In contrast, GP-POFA7 has denser structures and more spherical pores of unreacted particles due to the partial collapse of the geopolymer structures. The unreacted particles also impact the thermal stability of geopolymers. During exposure to high temperatures, these particles may expand or decompose, leading to internal stresses and microcracking within the matrix. This can reduce the thermal barrier capabilities of the material, as cracks and voids facilitate the transfer of heat through the material. According to Luo et al. (2022), the presence of unreacted particles can contribute to the development of microcracks during thermal cycles, weakening the material’s effectiveness as a thermal insulator [40].

**Figure 14:**
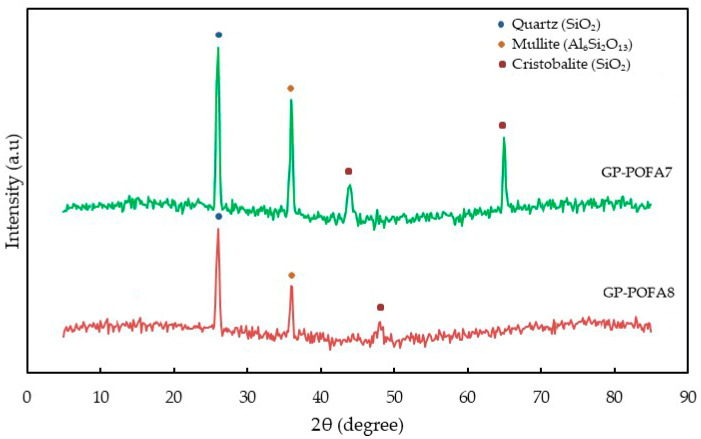
XRD patterns of GP-POFA8 and GP-POFA7.

The XRD analysis of GP-POFA7 and GP-POFA8 in Figure 14 reveals distinct crystalline phases that influence the material composition and thermal stability. The strong peak at 26°, identified as Quartz (SiO_2_), is more pronounced in GP-POFA7, indicating a higher content of unreacted silica. In contrast, GP-POFA8 exhibits a lower quartz peak, suggesting better geopolymerization and higher amorphous content. The presence of Mullite (Al_6_Si_2_O_13_) at 36° in both samples indicates high-temperature phase formation, contributing to structural integrity and enhanced fire resistance. However, GP-POFA8 demonstrates a more refined mullite phase, further improving its thermal stability.

The peaks observed at 45° and 65° in GP-POFA7 correspond to Cristobalite (SiO_2_), a high-temperature polymorph of silica that forms under prolonged heat exposure. Cristobalite, a crystalline form of silica (SiO_2_), undergoes a phase transition from its low-temperature α-phase to the high-temperature β-phase at approximately 200–270 °C. This transition is characterized by a significant volume change due to thermal expansion, which can induce internal stresses within materials containing cristobalite. Such stresses may lead to microcracking, particularly in ceramics and geopolymers, thereby compromising their structural integrity. Excessive amounts of cristobalite can reduce the flexibility of these materials, making them more susceptible to thermal shock and potential failure under rapid temperature fluctuations [41]. This is evident in the post-fire test of sample GP-POFA7, as shown in Figure 10, where the intumescent layer exhibits larger cracks and a more pronounced collapse at the center.

### 3.7. Thermal Stability Analysis

**Figure 15:**
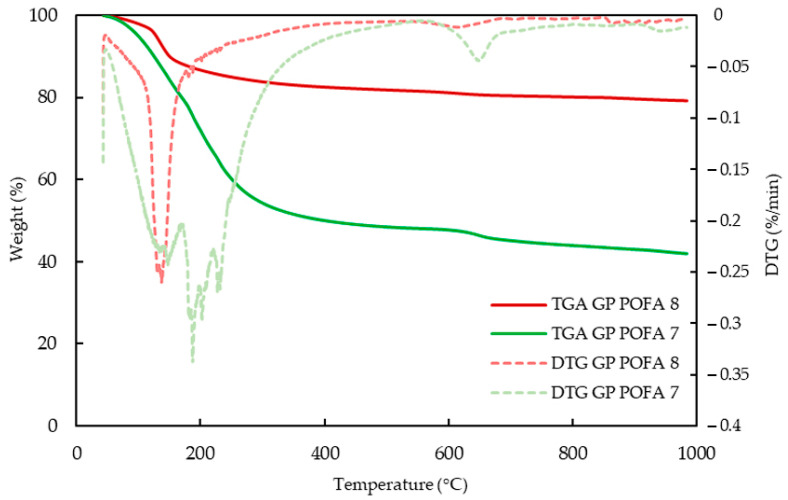
TGA thermogram and DTG curve of GP-POFA8 and GP-POFA7.

Figure 15 presents the weight loss behavior of two samples over a temperature range from 50 °C to 1000 °C, to compare the thermal stability and decomposition characteristics of two geopolymer formulations, GP-POFA8 and GP-POFA7.

The initial region, between 0 °C and 200 °C, shows a sharp decline in weight for both samples, indicating the loss of physically absorbed moisture and evaporation of free water within the geopolymer structures. This stage is typical for geopolymers, as their porous structure allows for water retention that is released upon heating. GP-POFA7 exhibits a more substantial weight loss in this region compared to GP-POFA8, suggesting that it has higher water content or more moisture trapped within its structure. This behavior is consistent with the findings of Temuujin et al. (2009), which noted that geopolymers with lower NaOH concentrations tend to retain more water, leading to greater weight loss in the early stages of TGA [42].

From 200 °C to 600 °C, both samples show a slower rate of weight loss, which corresponds to the dehydration of chemically bound water and the breakdown of less stable geopolymer bonds. The GP-POFA7 sample continues to lose weight more significantly than the GP-POFA8 sample, indicating a lower thermal stability. This could be due to a less dense or less polymerized structure, which is more susceptible to thermal decomposition. In contrast, the GP-POFA8 sample demonstrates greater resistance to weight loss, suggesting a more stable structure with stronger chemical bonds.

Beyond 600 °C, the weight loss stabilizes, with the GP-POFA8 sample retaining around 80% of its original mass, while the GP-POFA7 sample retains about 45% of its initial mass. The overall weight loss for GP-POFA8 and GP-POFA7 are 20.79% and 58.05%, which are equivalent to 11 mg and 6.14 mg, respectively. This phase represents the complete loss of water and decomposition of less stable components, leaving behind a more thermally stable residue. The higher residual mass in the GP-POFA8 sample indicates better retention of its structural integrity at elevated temperatures. This behavior is supported by studies by Kaze et al. (2021), which found that geopolymers with higher NaOH molarity and optimized curing conditions tend to form a more consolidated matrix, resulting in improved thermal resistance and lower weight loss during TGA [43].

The TGA/DTG analysis of GP-POFA7 revealed higher weight loss and sharper decomposition peaks, suggesting a material with more volatile components and unreacted crystalline phases. The maximum decomposition temperature (T_max_) for GP-POFA7 was observed at 187.4 °C, with an initial decomposition temperature (T_i_) of 89.9 °C, indicating its lower thermal stability. In contrast, GP-POFA8 exhibited a more gradual weight loss and lower-intensity decomposition peaks, reflecting better thermal stability and improved geopolymerization efficiency. This is supported by its T_max_ of 137.3 °C and T_i_ of 124.5 °C, highlighting its enhanced resistance to thermal decomposition.

## 4. Conclusions

This study evaluated POFA-based geopolymer coatings as a sustainable and high-performance fire-resistant material. By optimizing the POFA-to-alkaline activator (AA) ratio, NaOH molarity, and curing temperature, the best formulation (0.35 POFA-to-AA ratio, 8 M NaOH, 65 °C curing) significantly reduced heat transfer, achieving a temperature at equilibrium (TAE) of 151.2 °C. This represents a significant improvement over other tested formulations, underscoring the critical role of parameter control in determining the thermal performance of geopolymer coatings.

The microstructural analysis confirmed that the optimized coating formed a dense and uniform intumescent layer, enhancing thermal insulation and structural integrity. Thermogravimetric analysis (TGA) further demonstrated 80% mass retention beyond 600 °C, indicating superior thermal stability. The study also highlighted the importance of synthesis parameters, particularly NaOH concentration and curing temperature, in developing a stable geopolymer matrix.

These findings establish POFA-based geopolymer coatings as a sustainable and eco-friendly alternative to conventional fire-resistant materials, contributing to waste valorization and circular economy principles. Future research should focus on enhancing the mechanical properties, adhesion strength, and long-term durability of POFA-based coatings to ensure their widespread adoption in real-world applications. Investigating alternative curing methods, surface bonding improvements, and hybrid composite formulations could further expand its usability in extreme environments such as offshore structures, industrial plants, and aerospace components.

## Figures and Tables

**Table 1:** Chemical composition of POFA.

Element	SiO_2_	Al_2_O_3_	Fe_2_O_3_	CaO	MgO	SO_3_	Na_2_O	K_2_O	LOI
(wt.%)	57.42	4.27	3.99	11.01	4.19	0.95	3.79	6.27	8.11

**Table 2:** Mass of NaOH pellet to obtain 6 M, 8 M and 12 M of NaOH solution.

Concentration of NaOH, (M)	NaOH Mass (g)
6	120
8	160
12	240

**Table 3:** Uncoded levels selected for each factor.

Factors	Levels	
	Low	High
Concentration of NaOH (M)	6	12
Curing temperature (°C)	28	65
POFA: AA ratio	0.35	0.55

**Table 4:** Design matrix and response value for the fire resistance test.

Sample	POFA/AA	NaOH Molarity	Curing Temp. (°C)	TAE (°C)	TTAE (s)
GP-POFA1	0.55	8	65	184.8	1230
GP-POFA2	0.35	8	28	211.66	1196
GP-POFA3	0.35	6	45	192.37	1010
GP-POFA4	0.45	12	28	194.53	1117
GP-POFA5	0.45	8	45	190.97	654
GP-POFA6	0.45	6	65	177.58	1592
GP-POFA7	0.45	6	28	257.12	1109
GP-POFA8	0.35	8	65	151.16	995
GP-POFA9	0.55	6	45	208.02	442
GP-POFA10	0.45	8	45	191.32	671
GP-POFA11	0.45	12	65	237.51	1153
GP-POFA12	0.35	12	45	181.95	689
GP-POFA13	0.55	12	45	195.35	1115
GP-POFA14	0.45	8	45	191.59	689
GP-POFA15	0.55	8	28	208.46	546

**Table 5:** ANOVA analysis for TAE.

Source	Sum of Squares	DoF	Mean Square	F-Value	*p*-Value	
Model	8660.28	9	962.25	243.52	<0.0001	significant
A	452.60	1	452.60	114.54	0.0001	
B	30.70	1	30.70	7.77	0.0386	
C	748.60	1	748.60	189.45	<0.0001	
AB	0.93	1	0.9331	0.2361	0.6476	
AC	338.69	1	338.69	85.71	0.0002	
BC	4069.27	1	4069.27	1029.80	<0.0001	
A^2^	518.32	1	518.32	131.17	<0.0001	
B^2^	952.72	1	952.72	241.10	<0.0001	
C^2^	463.26	1	463.26	117.24	0.0001	
Residual	19.76	5	3.95			
Cor Total	8680.03	14				

DoF = Degree of Freedom, A = POFA/AA, B = NaOH molarity, C = Curing temperature.

**Table 6:** ANOVA analysis for TTAE.

Source	Sum of Squares	DoF	Mean Square	F-Value	*p*-Value	
Model	1,326,239.13	9	147,359.90	9.88	0.0106	significant
A	2769.12	1	2769.17	0.1857	0.6844	
B	1664.62	1	1664.62	0.1116	0.7518	
C	86,453.65	1	86,453.65	5.80	0.0610	
AB	261,638.89	1	261,638.89	17.55	0.0086	
AC	196,179.36	1	196,179.36	13.16	0.0151	
BC	52,763.19	1	52,763.19	3.54	0.1187	
A^2^	11,423.77	1	11,423.77	0.7662	0.4214	
B^2^	140,752.24	1	140,752.24	9.44	0.0277	
C^2^	488,756.52	1	488,756.52	32.78	0.0023	
Residual	74,548.62	5	14,909.72			
Cor Total	1,400,787.75	14				

DoF = Degree of Freedom, A = POFA/AA, B = NaOH molarity, C = Curing temperature.

**Table 7:** Experimental validation for fire resistance test.

	TAE (°C)			TTAE (s)		
	Experimental Value	Predicted Value	Error (%)	Experimental Value	Predicted Value	Error (%)
SV_1_	177.15	168.91	4.88	651	626	3.99
SV_2_	182.47	168.91	8.03	673	626	7.51
SV_3_	175.86	168.91	4.11	634	626	1.28
	Error		5.67	Error		4.26

## Data Availability

The original contributions presented in this study are included in the article. Further inquiries can be directed to the corresponding authors.
